# State-of-the-Art Estimation of Protein Model Accuracy Using AlphaFold

**DOI:** 10.1103/PhysRevLett.129.238101

**Published:** 2022-12-02

**Authors:** James P. Roney, Sergey Ovchinnikov

**Affiliations:** Harvard University, Cambridge, Massachusetts 02138, USA; John Harvard Distinguished Science Fellowship Program, Harvard University, Cambridge, Massachusetts 02138, USA

## Abstract

The problem of predicting a protein’s 3D structure from its primary amino acid sequence is a longstanding challenge in structural biology. Recently, approaches like alphafold have achieved remarkable performance on this task by combining deep learning techniques with coevolutionary data from multiple sequence alignments of related protein sequences. The use of coevolutionary information is critical to these models’ accuracy, and without it their predictive performance drops considerably. In living cells, however, the 3D structure of a protein is fully determined by its primary sequence and the biophysical laws that cause it to fold into a low-energy configuration. Thus, it should be possible to predict a protein’s structure from only its primary sequence by learning an approximate biophysical energy function. We provide evidence that alphafold has learned such an energy function, and uses coevolution data to solve the global search problem of finding a low-energy conformation. We demonstrate that alphafold’slearned energy function can be used to rank the quality of candidate protein structures with state-of-the-art accuracy, without using any coevolution data. Finally, we explore several applications of this energy function, including the prediction of protein structures without multiple sequence alignments.

Knowledge of 3D protein structures is critical for designing drugs, characterizing diseases, and creating a mechanistic understanding of cellular biology. Experimental approaches to protein structure determination can be costly and time consuming, so the ability to computationally predict protein structures from amino acid sequences is extremely useful. Recently, alphafold demonstrated breakthrough performance on protein structure prediction, with predictions often nearing experimental accuracy [1]. Approaches like alphafold have advanced the state-of-the-art in protein structure prediction by using deep learning methods to analyze coevolutionary information. To predict the structure of a target amino acid sequence, these methods first search a database of protein sequences to compile a multiple sequence alignment (MSA), which is essentially a collection of sequences that are evolutionarily related to the target sequence. MSAs are known to provide extremely useful information for predicting protein structures [2–4]. Intuitively, if two residues are in contact in a folded protein structure, mutations in the first position may induce a selective pressure for the second position to mutate. Such mutational covariance can be detected in MSAs, and this signal has been critical to the success of recent protein structure prediction models, including alphafold. However, the requirement of MSAs for protein structure prediction is sometimes problematic, since some proteins have few known homologs.

In theory, it should often be possible to predict protein structures without using MSAs, since protein structures are fully determined by their amino acid sequences [5]. More specifically, Anfinsen’s dogma states that protein structures fold to minimize free energy, which is a function of the protein’s 3D configuration and its amino acid sequence. Therefore, if one could model this energy function with sufficient accuracy, then one could predict protein structures by optimizing this function over the space of 3D configurations. Classical protein structure prediction methods like rosetta take this approach, and sample structures from a hand-designed energy function [6]. The challenge with this approach is twofold. First, it is difficult to accurately model the biophysical energy function that governs protein folding at a level of abstraction that is computationally tractable. Second, even with perfect knowledge of the energy function, there are an astronomically large number of possible protein geometries, so searching for the optimum is a difficult global optimization task [7].

Given the theoretical possibility of predicting protein structures without MSAs, it is interesting to speculate why alphafold remains dependent on MSAs for its accuracy. One intriguing possibility is that alphafold has learned an accurate energy function for scoring the accuracy of candidate protein structures, but the coevolutionary information in the MSA is necessary to locate an approximate global minimum in this energy function and circumvent the challenging optimization problem. After finding the neighborhood of the global minimum using the MSA, the later stages of the alphafold model may act as an “unrolled optimizer” and locally descend the learned energy surface to produce a refined structure prediction. alphafold also outputs various confidence scores related to the predicted accuracy of its structures, and these confidence scores may be determined by the value of its internal energy function. This hypothetical prediction mechanism is illustrated in Fig. 1. Note that we use the term “energy” to describe a function that has an optimum around the native structure and generally correlates with the probability that a protein sequence will adopt a given conformation, rather than a literal thermodynamic free energy. This notion of an energy function is reminiscent of energy-based machine learning models, which learn an unnormalized Boltzmann distribution to represent a target data density [8].

Our hypothesized prediction mechanism lends itself to experimental testing. Candidate structures can be supplied to alphafold as templates, which are used to incorporate known structural information from proteins that are related to the target sequence. In this Letter we show that, when a candidate structure is introduced as a template, alphafold’s confidence metrics are closely correlated with the actual accuracy of the candidate structure, even when no coevolutionary information is supplied. This suggests that alphafold has learned an accurate energy function for scoring protein structures that does not rely on coevolutionary information.

## Decoy scoring.—

Computational biologists have historically predicted protein structures based on related sequences with solved structures [11]. alphafold incorporates this approach by allowing the structures of up to four related proteins to be supplied to the model as templates. For each template, alphafold receives the template’s one-hot-encoded amino acid sequence, C*β* distance matrix, and backbone and side chain torsion angles as inputs. In addition, alphafold is given a mask indicating which atoms are unresolved in the template structure, and ignores torsion angles involving those atoms. Recent papers have demonstrated that alphafold’s template mechanism can be used to refine experimentally and computationally derived structural hypotheses [12,13].

We investigated whether alphafold has learned a coevolution-independent energy function for scoring protein structures by supplying alphafold with (i) a target amino acid sequence to be predicted and (ii) a “decoy structure” that is passed to the model as a template. The goal of this procedure is to score the plausibility of the target amino acid sequence adopting the geometry given by the decoy structure. It is motivated by the hypothesis that alphafold’s output structure will resemble the decoy introduced as a template and therefore, if alphafold has learned an accurate energy function that does not require coevolution information, the output confidence metrics will closely track the quality of the decoy. Note that no coevolutionary information is supplied to the model during this procedure.

We used a sequence of all “gap” tokens (which represent missing amino acids) to fill in the one-hot-encoded amino acid sequence associated with the decoy. We used the gap sequence due to an initial observation that high sequence identity between the decoy sequence and the target sequence caused alphafold to be overconfident in the decoy’s accuracy (Supplemental Material [14], Fig. S1). To keep the structural information supplied to alphafold from leaking the true decoy sequence, we masked out all side chain atoms aside from C*β*, and added a C*β* atom to all glycine residues (we decided to retain the C*β* atoms because alphafold uses a C*β* distance matrix to encode the template structure).

After processing its inputs, alphafold produces an output structure and two confidence metrics: the predicted template modeling score (pTM) score and the predicted Local Distance Difference Test (pLDDT) score [18,19]. To determine whether alphafold has learned a MSA-free energy function for assessing protein structure accuracy, we investigated whether we could accurately rank the decoy structures based on alphafold’s outputs. For each decoy, we computed a “composite confidence score” by multiplying the output pLDDT, the output pTM, and the TMscore between the decoy structure and the alphafold output structure. The last term adjusts for the fact that alphaf’s confidence metrics ultimately reflect the accuracy of the output structure (which can differ from the decoy structure), while we were interested in scoring the decoy structures for the sake of direct comparison with other decoy-ranking methods.

## ROSETTA decoys.—

Using the procedure outlined above, we aimed to determine whether AlphaFold’s outputs could be used to assess the accuracy of decoy structures introduced as templates. For our initial evaluation we used the rosetta decoy dataset, which contains 133 native protein structures (targets) with thousands of decoys for each native structure [20]. We compared alphafold’s ability to assess the quality of decoy structures with the rosetta energy function, as well as DeepAccNet, which is a state-of-the-art machine learning model for estimating the accuracy of protein structure models [21]. All reported results are from alphafold model 1 with one recycling iteration.

**FIG. 2.**
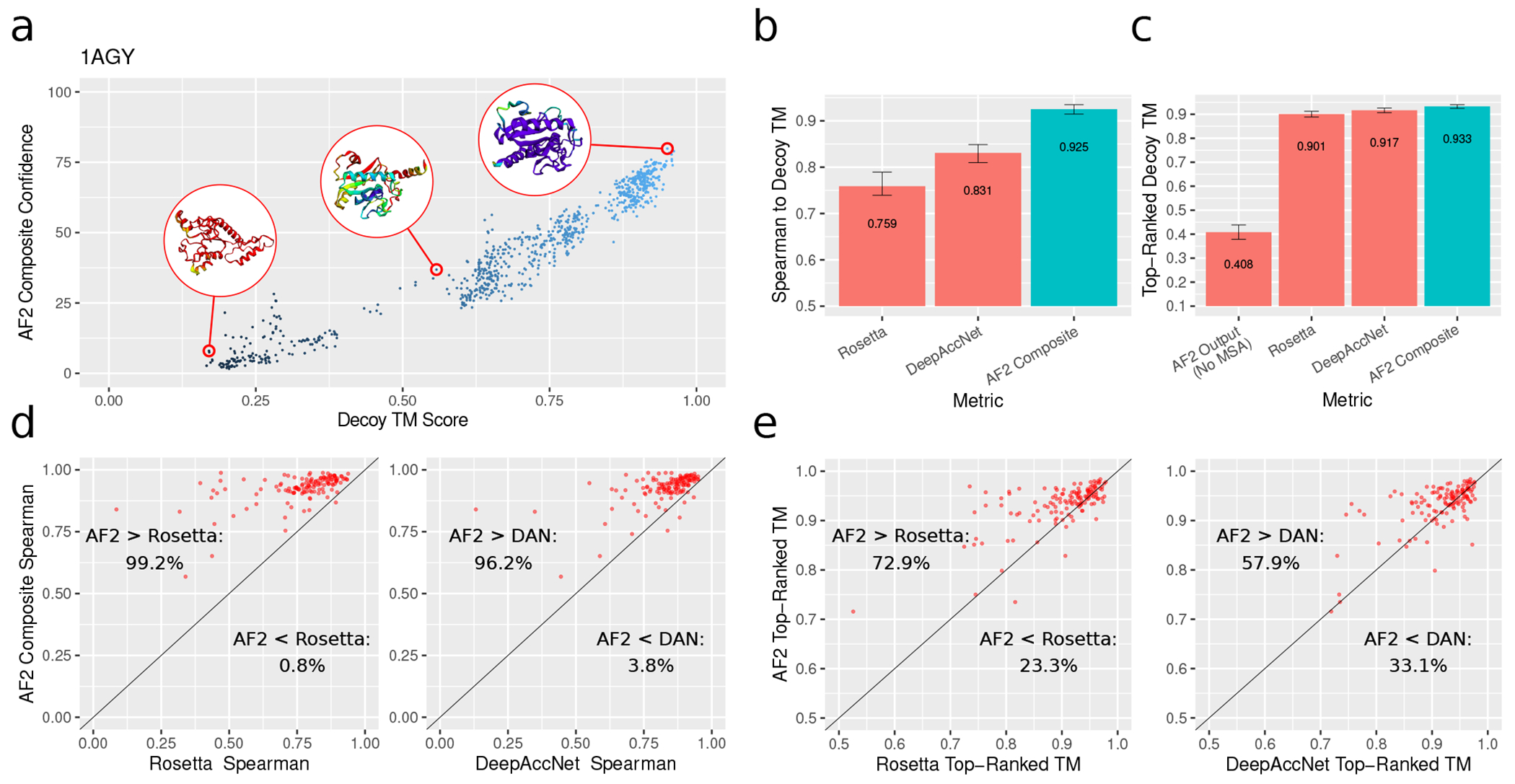
Decoy ranking results on the rosetta decoy dataset. (a) Decoy TM score vs composite confidence for an example target. Three selected alphafold output structures are visualized, color indicates model confidence. (b) Mean Spearman correlations between various metrics and decoy TM Score. (c) Mean TM Scores of the top-ranked decoys for various metrics, as well as the mean TM Score of alphafold’s prediction with no MSA. All error bars in (b) and (c) are bootstrap 95% confidence intervals of the mean. (d) Comparison of Spearman correlations for alphafold and rosetta (left) or DeepAccNet (right). (e) Comparison of top-1 accuracies for alphafold and rosetta (left) or DeepAccNet (right). For (d) and (e), each dot is a target in the rosetta decoy dataset; a dot’s position in each scatterplot depicts the relevant Spearman correlation or top-1 accuracy values computed over the decoys corresponding to that target.

We found the correlation between the composite confidence score and decoy quality to be robust and consistent. The average Spearman rank correlation between the composite confidence score and the quality of the decoy (as measured by TM Score to the native structure) was 0.925, compared to average correlations of 0.831 and 0.760 for DeepAccNet and the rosetta energy function. Another practical indicator of decoy-ranking performance is the quality of the top-ranked decoy for each target. on the rosetta decoy dataset, the top-ranked decoys selected via the composite alphafold confidence score had an average TM Score of 0.933 compared to 0.917 for DeepAccNet and 0.901 for rosetta. More details on the rosetta dataset are given in Fig. 2.

Overall, these evaluations indicate that alphafold can assess the quality of candidate protein structures with state-of-the-art accuracy, even when no coevolution information is provided. It should be noted that alphafold’s structure predictions were of low quality when no templates were provided (average TM score of 0.408). Yet despite being unable to predict the structures of these proteins without a MSA, alphafold achieved excellent performance assessing the quality of decoys without any MSA inputs. This provides evidence for the hypothesis that alphafold has learned an energy function that is largely independent of coevolution information, but needs coevolution information to search for global optima in this energy landscape.

## casp14.—

To assess the decoy-ranking ability of alphafold on a novel sample of proteins, we performed an additional evaluation on the estimation of model accuracy (EMA) task from casp14 [22]. To set up the casp14 EMA experiment, the CASP organizers created a set of decoy structures by taking the 150 most accurate server submissions for each structure prediction target in casp14. Note that the decoy set does not include predictions from alphafold, since alphafold was entered in casp14 as a human group rather than a server. We replicated this evaluation using alphafold (with the gap sequence) to assess the decoy structures, and compared the results with ranking methods entered in casp14.

**FIG. 3.**
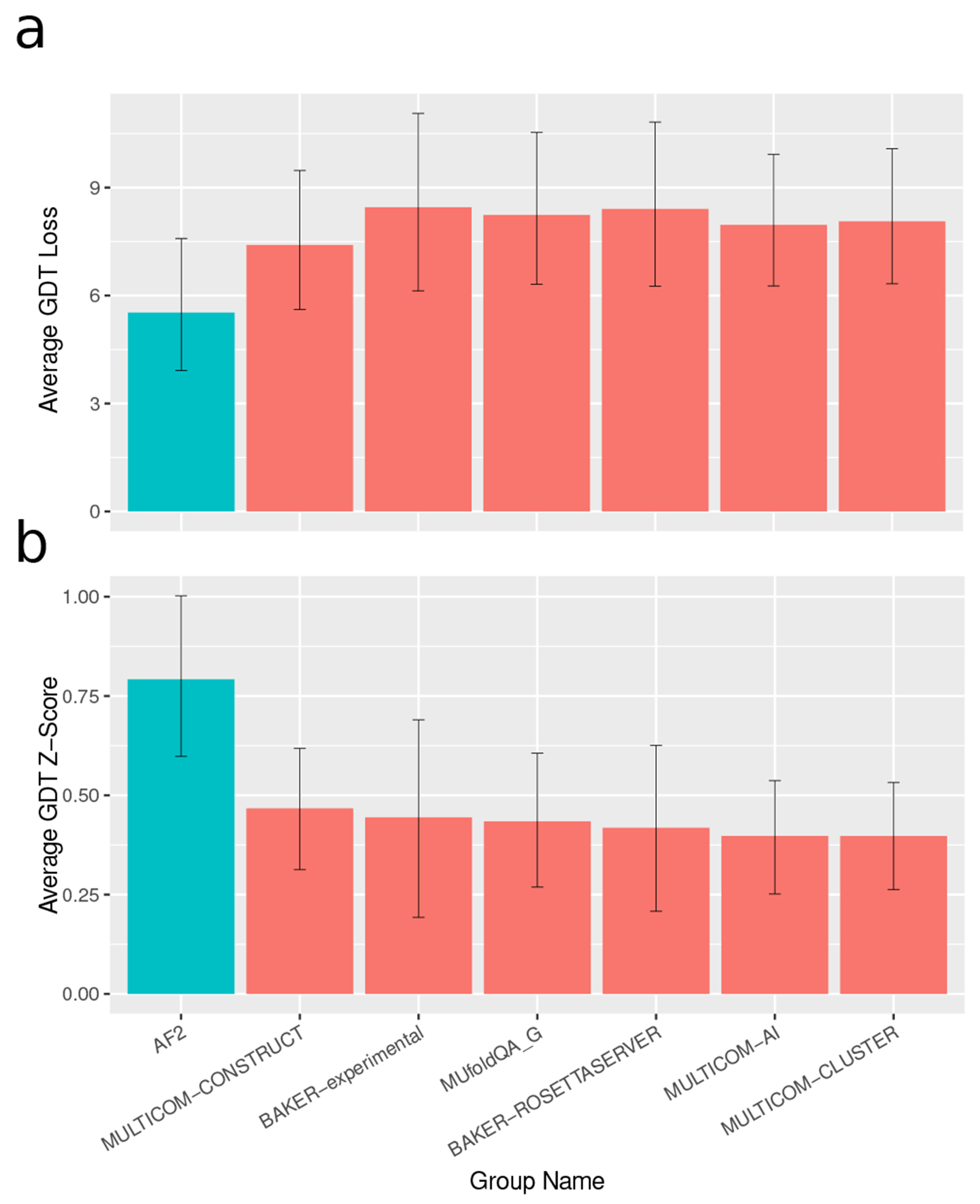
Decoy ranking results on CASP. (a) GDT_TS loss for alphafold and top EMA methods from casp14. (b) GDT_TS *Z*-scores for alphafold and top EMA methods from casp14. Error bars are bootstrap 95% confidence intervals of the mean.

The CASP assessors evaluated EMA methods based on their top-1 GDT_TS loss, which is the difference in GDT_TS scores between the best decoy and the top-ranked decoy by a given EMA method [23]. EMA methods were ranked based on their average GDT_TS loss over targets where at least one decoy had GDT_TS over 0.4, as well as the average *Z*-score of their GDT_TS loss over these targets. For both metrics, the alphafold composite confidence score significantly outperformed all other EMA methods entered in casp14. Results from the casp14 evaluation are presented in Fig. 3.

These results indicate that alphafold can reliably assess the accuracy of candidate protein structures without the use of coevolution information. However, coevolution data (or a method that can generate decoys close to the correct structure) are still necessary for accurate structure prediction, since alphafold generally fails to predict accurate structures for the casp14 targets without a MSA (Fig. S3).

## Applications.—

Our finding that alphafold can assess the accuracy of candidate protein structures without the need for coevolution data opens up several exciting applications. One such application is the prediction of protein structures without MSAs. In theory, it should be possible to accurately predict protein structures by searching over the space of possible decoy structures and finding those that are highest ranked by alphafold. However, given the vast number of possible candidate structures, an exhaustive search is intractable.

**FIG. 4.**
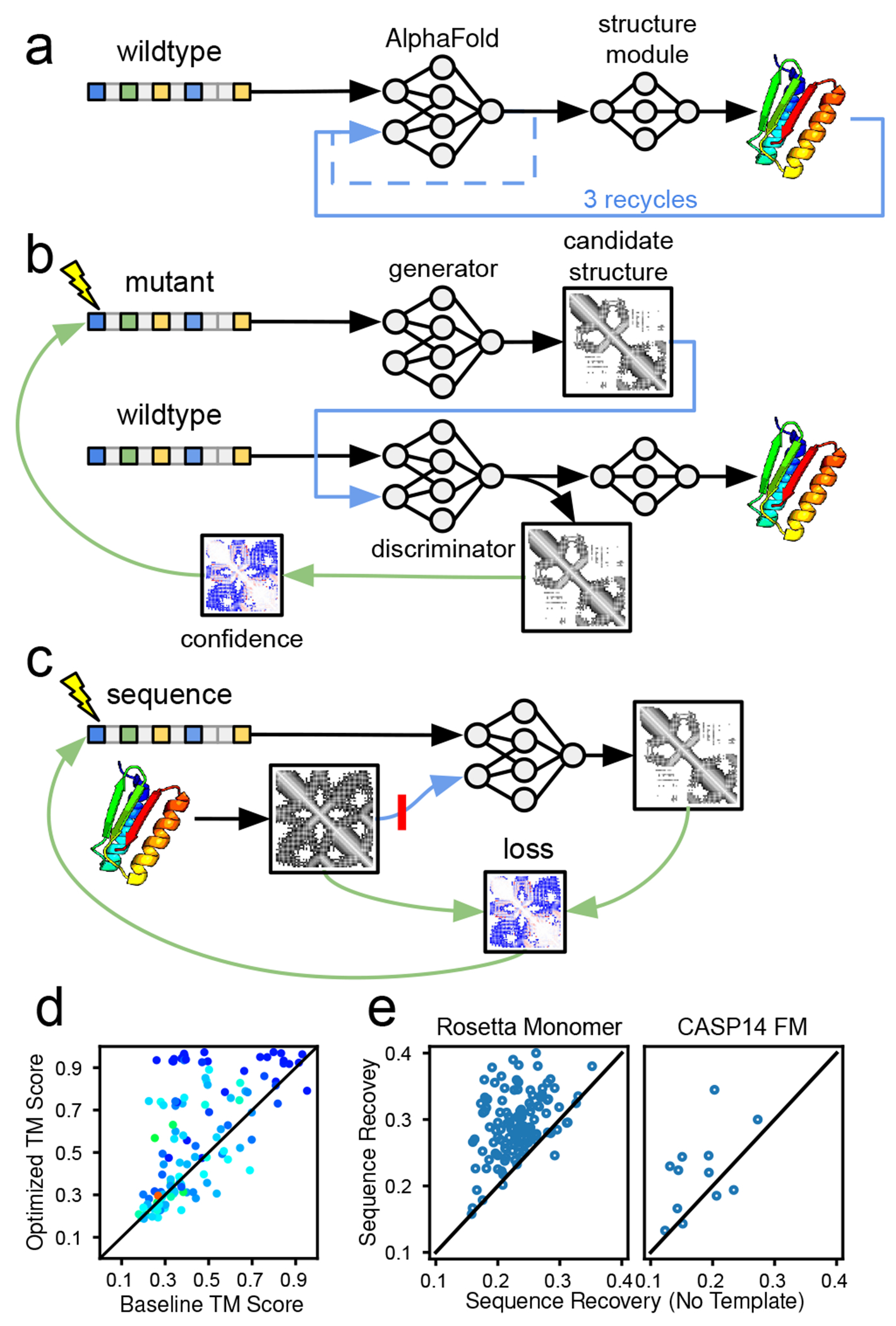
Application of alphafold’s template mechanism for sequence and structure generation. We compare single-sequence structure prediction with (a) the baseline structure prediction protocol (alphafold with a single-sequence input and three recycles) or (b) two instances of alphafold for structure generation and discrimination. (c) Protocol for sequence design to minimize loss between desired and predicted structure via distogram, with and without template (red line). (d) Comparing structure accuracy of (a) vs (b) on the rosetta decoy set. Dots colored by PLDDT red to blue (50 to 90). (e) Comparing sequence recovery with and without templates on the rosetta monomeric and casp14 FM datasets.

One way of mitigating this intractability is to search over the output space of a generative model of realistic protein structures. Instead of training a new generative model of candidate structures, we designed a generator-discriminator pipeline that links two instances of alphafold [Fig. 4(b)]. The first instance of alphafold (the generator) takes an arbitrary amino acid sequence as input, and produces a candidate protein structure as output. This candidate structure is then supplied to the discriminator as a template (with a sequence of gap tokens). Finally, the discriminator tries to predict the structure of the target sequence using the template, and produces confidence outputs in the process. As demonstrated by our previous experiments, these confidence metrics are strongly correlated with the accuracy of the candidate structure. By perturbing the input sequence to the generator, we can explore the space of candidate structures while using the discriminator’s confidence metrics as an indicator of accuracy. We performed this exploration by backpropagating the discriminator’s confidence signal to the input sequence and updating it via gradient ascent, thereby molding the input sequence to produce a high-quality candidate structure from the generator.

Using this approach we were able to improve upon alphafold’s structure predictions when no MSAs were available. Although we did not use recycling in the generator and discriminator models, we compared the quality of our optimized predictions to a baseline created by running alphafold with a single sequence input and three recycling iterations. On the rosetta decoy set, we could significantly improve prediction quality (ΔTM score > 0.1) on 50 examples out of 123 compared to running the three-recycle baseline [Fig. 4(d)]. We hypothesize that this procedure is able to improve alphafold’s predictions because it performs a more wide-ranging search than the “unrolled optimizer” implemented by alphafold itself. Though our results demonstrate the potential of searching over candidate structures using alphafold’s learned energy function, the current optimization protocol sometimes gets stuck in local minima with low accuracies and low confidence scores (Figs. S11–13).

Since alphafold’s learned energy function can determine the level of compatibility between a protein sequence and structure without the need for coevolution data, it is potentially applicable to protein design (i.e., the problem of finding a protein sequence that folds into a target backbone geometry). Our Letter suggests a straightforward approach to protein design using alphafold: supply the desired backbone structure to alphafold as a template (with a sequence of gap tokens and side chains masked), and optimize the composite confidence score with respect to the input sequence. To facilitate gradient-based optimization, we used the categorical cross entropy between alphafold’s predicted distance matrix and the template distance matrix as a surrogate loss for the composite confidence score. Using this loss circumvents the need to differentiate the TM score, which involves an iterative alignment procedure with potentially unstable gradients. The cross entropy loss can replace the entire composite score (including the pTM and pLDDT components), because when alphafold’s confidence in its output structure is low its predicted distance distributions become wider, thereby increasing the cross entropy. Pairing our target backbones with sequences designed by cross entropy optimization resulted in higher composite confidence scores than pairing them with their native sequences, indicating that optimizing the cross entropy loss effectively optimizes the composite confidence as well (Fig. S9).

Fixed-backbone protein design methods are often benchmarked based on the average fraction of residues that match between the designed sequence and the true native sequence for the target backbone [24]. Our design procedure achieved an average sequence recovery of 29.1% on the rosetta decoy dataset, which is comparable to energy-based design methods like rosetta [25].

Repeating the same procedure without a template input resulted in significantly lower sequence recovery [Fig. 4(e)]. This is likely because, without a template or MSA, alphafold often fails to predict the correct structure for the input sequence. This leads to “false negatives” while optimizing the input sequence to match the target backbone (i.e., input sequences that would actually fold into the target backbone are mispredicted by alphafold and incorrectly assigned high loss). The template input eliminates false negatives by providing a good starting point for alphafold’s structural optimization, allowing alphafold to confidently and accurately predict when an input sequence will fold into the target structure. The effectiveness of template inputs at increasing sequence recovery supports our hypothesis that alphafold has learned an energy function that can assess sequence-structure agreement, but needs coevolution data or templates to help search for optimal structures.

## Conclusions.—

In this Letter we have provided evidence that alphafold has learned a protein structure energy function that does not need coevolution information to achieve high accuracy, although alphafold still needs coevolution data to search for global minima in this function. This finding has significance for the interpretation of protein structure prediction models, as well as practical applications. These applications include the prediction of protein structures when MSAs are not available and the improvement of protein design methods.

The code used to run the evaluations in the Letter, as well as the raw data, is available at [26].

## Supplementary Material

SI

## Figures and Tables

**FIG. 1.**
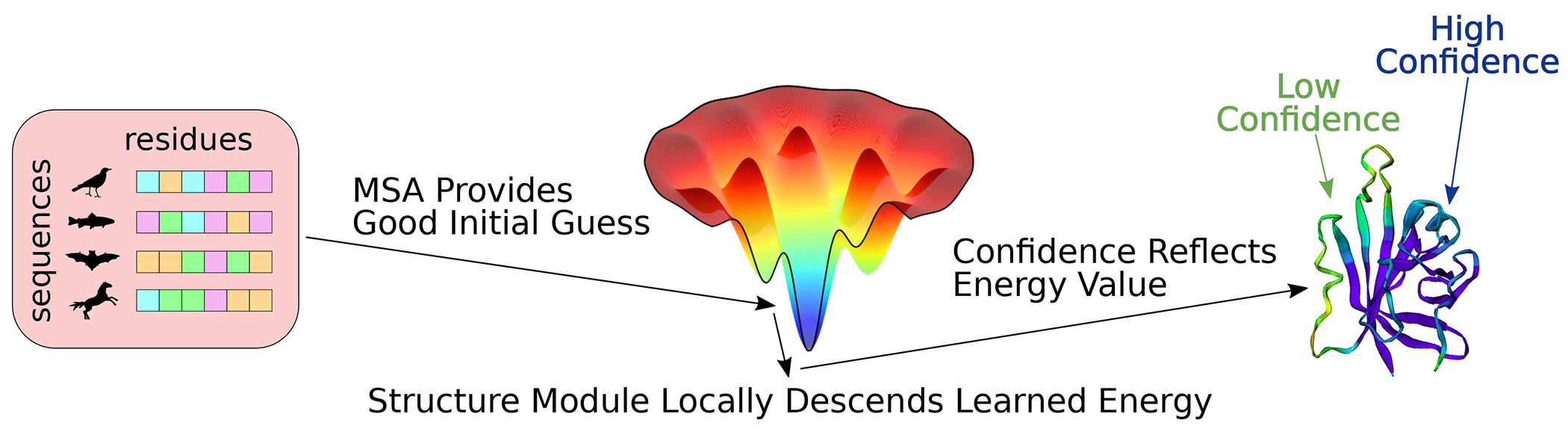
The hypothesized role of coevolutionary information in alphaf’s predictions. Images inspired by [9,10].
