# Supplementary material for: State-of-the-Art Estimation of Protein Model Accuracy Using AlphaFold
Source: Phys Rev Lett. Author manuscript; Available in PMC 2025 Jun 19. (PMC12178128; doi:10.1103/PhysRevLett.129.238101)
Supplement: SI [file NIHMS2044141-supplement-SI.pdf]

State-of-the-art estimation of protein model accuracy using AlphaFold: Supplemental Information

James P. Roney* and Sergey Ovchinnikov†

I. COMPARISON OF DECOY SEQUENCES

As mentioned in the main text, we investigated two choices for the one-hot encoded sequence associated with the decoy structure: the target amino acid sequence, and a sequence of "gap" tokens. While AlphaFold's confidence metrics are robustly correlated with decoy quality when using the gap sequence, this correlation is much lower when using the target sequence. A comparison of decoy-ranking performance for each of the two sequences on an example protein is given is Figure S1.

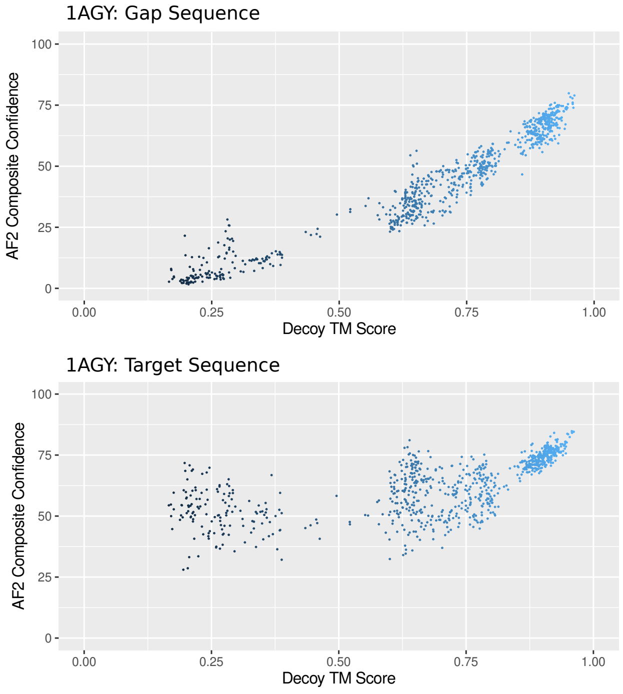

FIG. S1. Correlation between AlphaFold's confidence metrics and decoy quality when using the gap sequence (top) and the target sequence (bottom).

We hypothesize that, when using the target sequence, AlphaFold is overconfident in the quality of the decoy structures due to the high sequence identity between the decoy and the target. Due to this difference in performance, we used the gap sequence in all of our evaluations.

II. ANALYSIS OF OUTPUT STRUCTURES

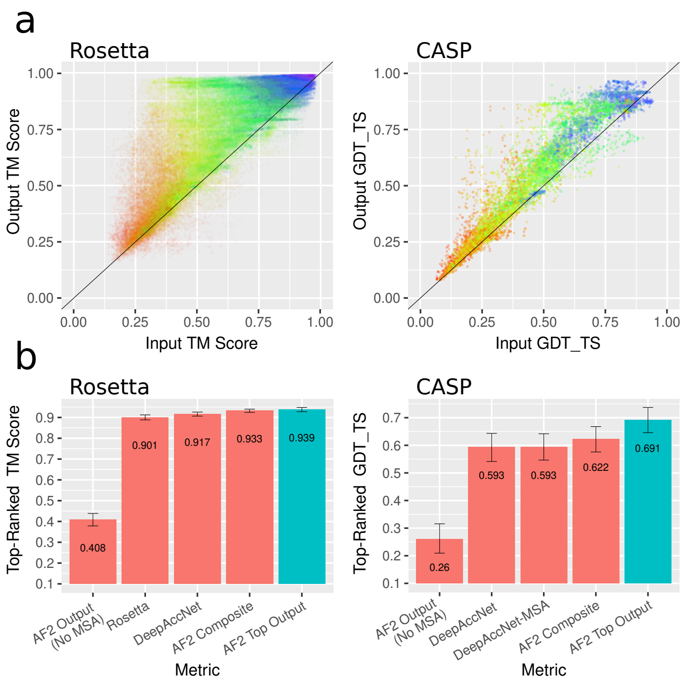

FIG. S2. Comparison between input and output structure qualities. (a) TM Score / GTS_TS of AlphaFold output structure vs. TM Score / GDT_TS of decoy structure supplied as a template. Each dot is single decoy in the Rosetta decoy set (left) or CASP EMA set (right), color indicates composite confidence score. (b) Mean TM Scores of the top-ranked Rosetta (left) and CASP (right) decoys for various ranking metrics, including the AlphaFold output structures with the highest pLDDT × pTM product in blue. Error bars are bootstrap 95% confidence intervals of the mean.

AlphaFold's output structures can differ from the structures provided as templates. Figure S2 illustrates that AlphaFold's output structures are often similar in quality to the decoy structures, and sometimes are substantially improved in terms of TM Score and GDT_TS. This necessitates the use of a term in the AlphaFold composite score that tracks how much the AlphaFold output structure changes from the decoy structure, since AlphaFold's confidence metrics ultimately reflect the accuracy of the output structure. As illustrated in Figure

* jamesproney@gmail.com; Harvard College, Cambridge, MA, USA
† so@fas.harvard.edu; John Harvard Distinguished Science Fellowship Program, Harvard University, Cambridge, MA, USA

S2, applying this correction causes the confidence score to track the quality of the input (i.e., the color gradient in Figure S2A progresses along the x-axis) rather than the quality of the output (the y-axis). While AlphaFold is sometimes capable of improving decoy structures without coevolution information, it generally fails to predict these structures from scratch when no coevolution information is provided. This supports the idea that AlphaFold can perform local optimization over its learned energy function, but needs coevolution data or a template to locate a good starting point for this optimization.

III. CASP EVALUATION EXTENDED RESULTS

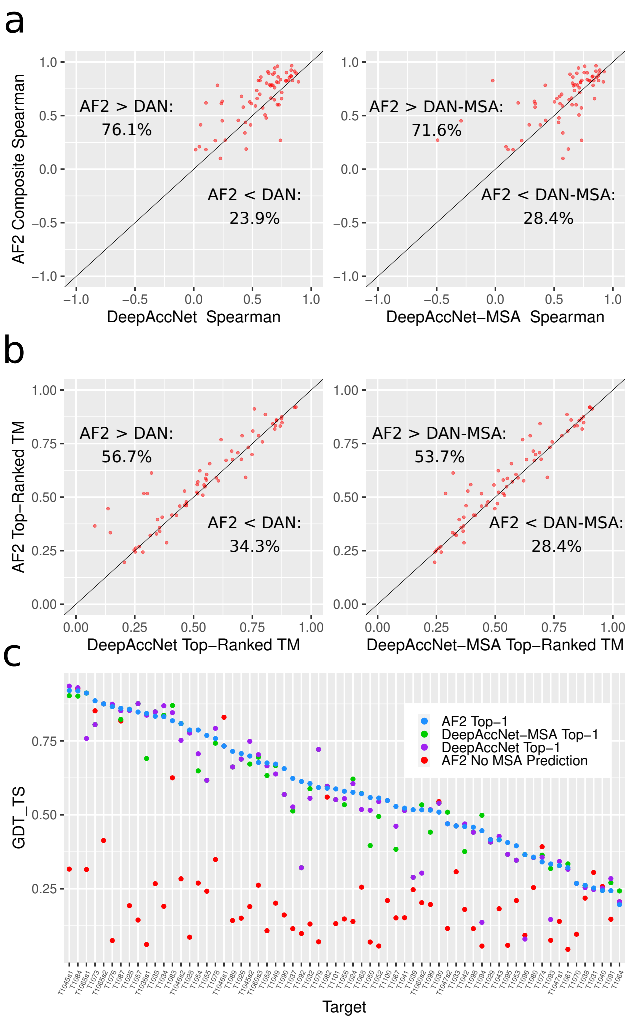

FIG. S3. Extended Results from the CASP14 Evalutation. (a) Comparison of Spearman correlations for AlphaFold and DeepAccNet/DeepAccNet-MSA. (b) Comparison of top-1 accuracies for AlphaFold and DeepAccNet/DeepAccNet-MSA. (c) Top-1 results on each CASP14 target for AlphaFold, DeepAccNet, and DeepAccNet-MSA, as well as the MSA-free AlphaFold prediction for each target.

As described in the main text, AlphaFold outperforms all methods from the CASP14 Estimation of Model Accuracy experiment (according to the metrics used by CASP). In this section we give more details on the results of the CASP evaluation. In particular, we specifically compare AlphaFold to DeepAccNet (entered in CASP14 as BAKER-ROSETTASERVER) and DeepAccNet-MSA (entered as BAKER-Experimental), which are two of the top-performing methods from the CASP14 EMA experiment. As shown in figure S3, AlphaFold outperforms both of these methods on a majority of targets.

AlphaFold can reliably assess the accuracy of candidate protein structures without the use of coevolution information. However, coevolution data (or a method that can generate decoys close to the correct structure) are still necessary for accurate structure prediction. When AlphaFold is tasked with predicting the CASP14 targets without any MSA inputs, its structure predictions are generally much less accurate than the top-ranked decoy based on AlphaFold's confidence metrics (Figure S3C).

In the main text, we used the AlphaFold composite confidence score with a "gap" decoy sequence to rank the CASP decoys. We chose this configuration because it gave the best performance on the Rosetta dataset, which we used for validation. For completeness, Figure S4 shows the performance of other variations of the AlphaFold ranking scheme on the CASP dataset. In particular, we also tried using a sequence of all alanine residues, as well as using only the pLDDT and pTM scores in the composite confidence score. All of these configurations gave state-of-the-art results.

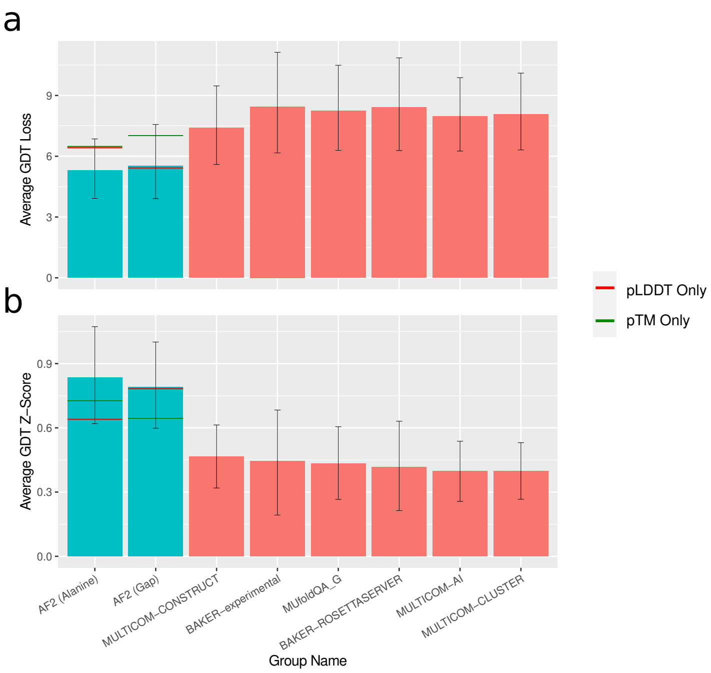

FIG. S4. CASP14 performance for several variations of the AlphaFold ranking system. (a) GDT_TS Loss (b) GDT_TS Z-Scores.

IV. ADDITIONAL ROSETTA RANKING RESULTS

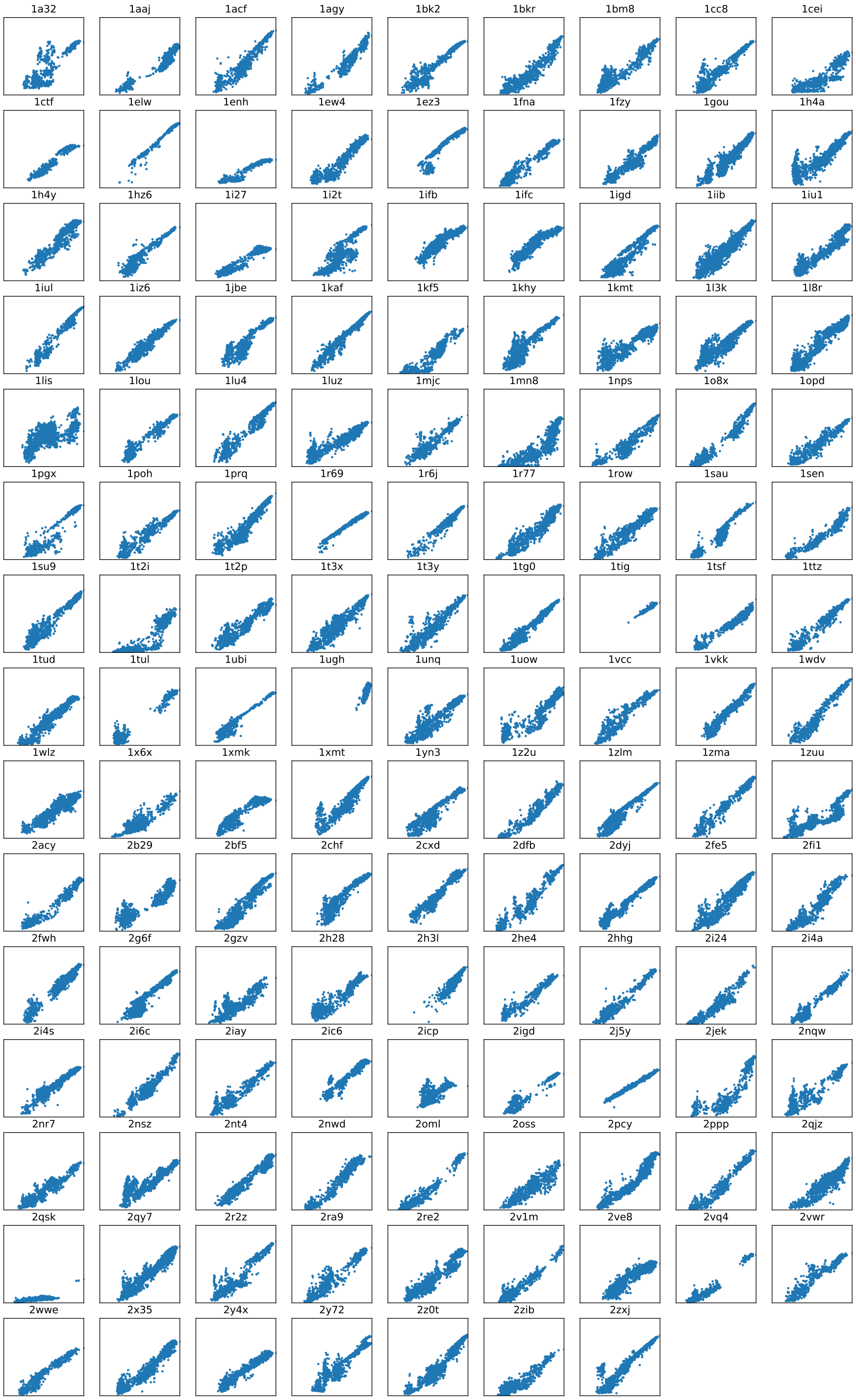

FIG. S5. AF2 composite confidence vs. decoy TM Score for all targets in the Rosetta Decoy set.

V. MUTANT EFFECT PREDICTION

Our results provide evidence that AlphaFold has learned an energy function which is capable of assessing the accuracy of candidate protein structures without the need for coevolution information. A natural question is whether this energy function is capable of predicting the effects of point mutations on protein structure and stability. As a preliminary exploration of this question, we attempted to use AlphaFold's confidence metrics to predict the effects of point mutations on the fitness of β-Lactamase and a ParDE toxin-antitoxin complex, as measured by deep mutation scans. For β-Lactamase, we used the dataset from [23], which contains fitness assays for all point mutations to β-Lactamase. For the toxin-antitoxin complex, we used the dataset from [24], which contains fitness scores for a total of 9194 mutant variants at 4 positions of the toxin-antitoxin interface.

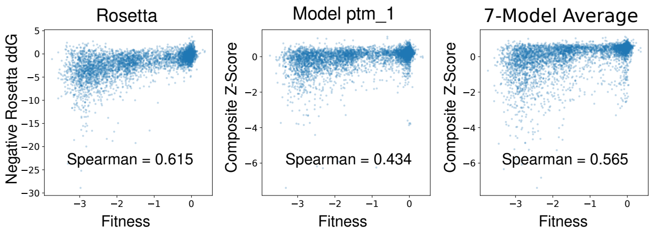

FIG. S6. Correlation between β-Lactamase mutant fitness and Rosetta $\Delta\Delta$G (left) composite confidence from AlphaFold pTM model 1 (center) and the average composite confidence from 7 AlphaFold models (right).

To predict the fitness effect of a given point mutation, we supplied AlphaFold with the native structure as a template (with sidechains masked and a gap sequence). For each mutation, we set the target sequence to the mutated sequence and recorded the model's composite confidence score. Based on our previous results, we might expect that the introduction of a destabilizing mutation into the target sequence will make it less compatible with the native structure supplied as a template, resulting in a drop in confidence. We used PDB 1XPB as the native structure for β-Lactamase, and chains A and D of PDB 5CEG for the toxin-antitoxin complex (chain D was trimmed to the interacting region of positions 49 through 87 to speed up computation). On the β-Lactamase system, we also used the Rosetta $\Delta\Delta$G values computed by [25] as a physics-based baseline for mutation effect prediction.

For β-Lactamase, we found that AlphaFold's composite confidence achieved a relatively low Spearman correlation with fitness measurements of 0.43. However, the AlphaFold composite score achieved a higher correlation of 0.56 when using an ensemble of seven AlphaFold models (two from AlphaFold-ptm and five from AlphaFold-multimer, Table S1). To create the ensemble we averaged the z-scores of each model's composite confidence. The Rosetta $\Delta\Delta$G values had an even higher correlation of 0.62 (Figure S6). This result suggests that, while AlphaFold's learned energy function is more accurate than Rosetta at predicting the global accuracy of protein structures, Rosetta is better at predicting the impacts of point mutations. This is not altogether unsurprising, since AlphaFold was trained on stable structures, so its learned energy may be less sensitive to the effects of single destabilizing mutations and better suited to analyzing more global features of the sequence and structure.

Model	Single	Average
ptm_1	0.43	0.43
ptm_2	0.44	0.46
mult_1	0.50	0.49
mult_2	0.57	0.54
mult_3	0.53	0.55
mult_4	0.58	0.57
mult_5	0.40	0.56

TABLE S1. Spearman correlation between AlphaFold composite confidence and β-Lactamase mutant fitness when averaging increasingly large ensembles of AlphaFold models. "Average" denotes an ensemble of all of the models preceding each row.

For the toxin-antitoxin dataset, the authors suggest treating the data as binary outcomes with fitness measurements above 0.1 being considered "fit". Using this cutoff, we computed AlphaFold's "top-k success rate" as the fraction of fit mutations among the k highest-ranked mutations according to AlphaFold composite confidence. To summarize the success rate of a given model, we computed a "success rate AUC" (sAUC) by averaging all of the top-k success rates with k ranging from 1 to 500. Compared with the traditional ROC curve, this metric better reflects the hypothetical experimental fitness rate of computationally-generated variant libraries. We also report the typical ROC AUC metric. The toxin-antitoxin results are presented in Table S2 and Figure S7. These results indicate that AlphaFold's composite confidence generally succeeds in filtering out fit mutations, and that larger ensembles of models perform better.

	sAUC		ROC AUC		Spearman	
Model	Single	Avg	Single	Avg	Single	Avg
ptm_1	0.72	0.72	0.89	0.89	0.37	0.37
ptm_2	0.29	0.47	0.83	0.88	0.32	0.36
mult_1	0.85	0.72	0.91	0.90	0.38	0.38
mult_2	0.79	0.79	0.94	0.93	0.36	0.39
mult_3	0.81	0.82	0.94	0.94	0.40	0.40
mult_4	0.80	0.83	0.90	0.94	0.29	0.39
mult_5	0.83	0.84	0.92	0.94	0.37	0.39

TABLE S2. Success rate AUC, ROC AUC, and Spearman correlation between AlphaFold composite confidence and toxin-antitoxin mutant fitness when averaging increasingly large ensembles of AlphaFold models. "Average" denotes an ensemble of all of the models preceding each row.

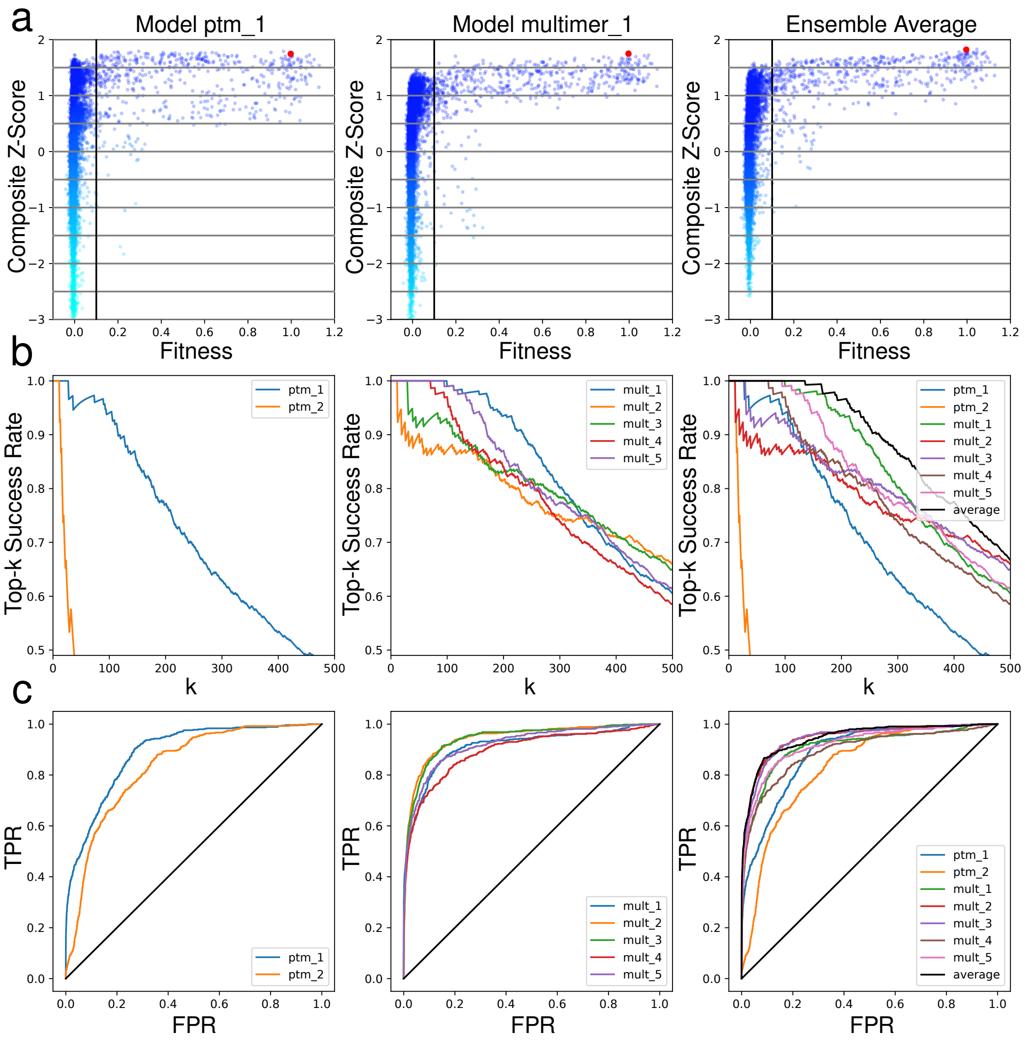

FIG. S7. Correlation between AlphaFold confidence metrics and the fitness of a ParDE toxin-antitoxin complex. (a) Plot of mutant fitness vs. AF2 composite z-score for AlphaFold pTM model 1 (left), AlphaFold multimer model 1 (center), and an average of 7 AlphaFold models (right). The vertical line represents the cutoff for a mutated sequence to be considered "fit," and the red dot represents the native sequence. (b) Top-k success rates for the detection of fit mutants from AlphaFold confidence scores, with k ranging from 1 to 500. (c) ROC curves for the detection of fit mutants from AlphaFold confidence scores.

VI. PROTEIN DESIGN EXPERIMENTS

As described in the main text, we applied AlphaFold's learned energy function to design protein sequences that fold into a set of target backbone structures. For each target structure, AfDesign was used to generate sequences that AlphaFold predicts will fold into the desired structure (available at `https://github.com/sokrypton/ColabDesign`). For this task, we used the `fixbb` (fixed backbone) protocol and the 3-stage design method. This protocol uses a categorical cross entropy loss between the desired and the predicted distogram (a distogram is a tensor that contains a binned distribution of distances for every pair of residues). The protocol optimizes this loss over an input sequence (encoded as a $N \times 20$ matrix) that is used as input for both the target features and MSA features of AlphaFold.

During the 3-stage design protocol, the sequence-matrix is initially an unconstrained and continuous set of logits. Over time, the sequence-matrix is gradually constrained to become a normalized probability distribution according to the formula

`(1-p) * logits + p * softmax(logit/temp)`.

For the first 300 iterations `p` is linearly scaled from 0 to 1, resulting in a softmax distribution at stage 2. For the next 200 iterations the temperature is reduced from 1.0 to 0.01, so the sequence-matrix approaches a one-hot encoded sequence. At the third stage, the one-hot encoded sequence is directly optimized for 50 steps, using the straight-through estimator. At each step of optimization (across all 3 stages), only one AlphaFold model is used, but the model parameters are randomly selected from either model 1 or 2 (since these are the only ones trained with template inputs). To help the optimizer es-

cape local minima, dropouts are enabled throughout the model. When a template input is used, 15% of the sites are randomly dropped at each iteration. At the third stage, the dropouts are disabled and the sequence with best loss is selected as the final design. Using 5 random seeds, this design protocol was repeated 5 times, with and without template inputs, to generate a total of 10 sequences. The results reported are the average of 5 runs.

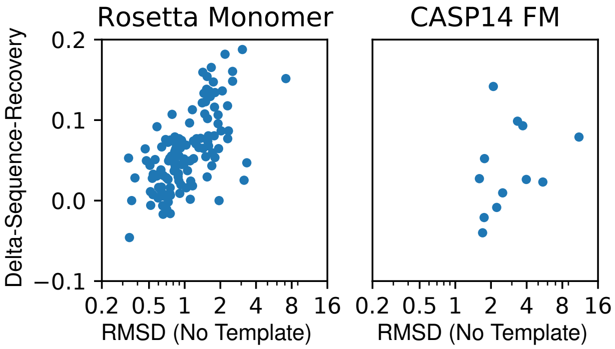

FIG. S8. In cases where sequence optimization was relatively unsuccessful (i.e., the designed sequence still had high predicted RMSD with the target backbone), supplying the target backbone as a template improved sequence recovery.

As depicted in Figure 4E, we found that utilizing a template resulted in substantially higher levels of native sequence recovery. This improvement was especially large in cases where template-free optimization failed to find an input sequence that folded closely into the target backbone geometry (Figure S8).

Sequences were generated for both the proteins in the Rosetta decoy and CASP14 datasets. For CASP14, domains labelled as FM (free-modeling) were selected, as these are significantly different from any proteins in the AlphaFold training set. To reduce runtime, only proteins/domains of length 150 or less were used.

While our design experiments utilized a cross entropy loss to facilitate smoother optimization, we would ultimately like to optimize the composite confidence score between the designed sequence and the target backbone (i.e., the composite score returned when the target backbone is supplied as a gap-encoded template and the designed sequence is supplied as the target sequence). To confirm that optimizing our surrogate loss function resulted in optimized composite confidence scores, we compared the composite confidence scores between our target backbones and their designed sequences with the composite scores between the target backbones and their wildtype sequences. We found that the composite scores between the backbones and the designed sequences were higher. Our decoy-ranking experiments showed that the composite confidence score between a given structure and its wildtype sequence is generally already very high, so this result indicates that optimizing the cross entropy loss resulted in the effective optimization of the composite confidence score (Figure S9).

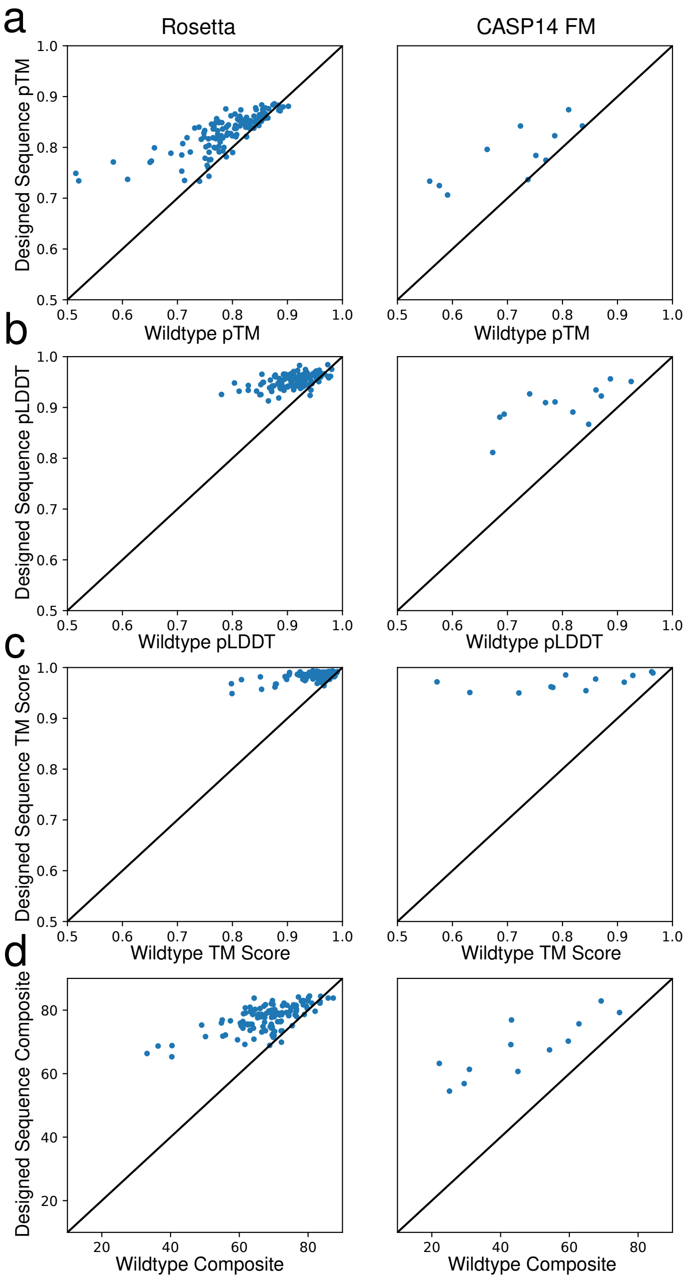

FIG. S9. The AlphaFold composite confidence score (and its individual components) is higher for designed sequences than for wildtype sequences on both the Rosetta (left) and CASP14 (right) datasets. (a) AlphaFold pTM for each backbone template when using the designed sequence as the target sequence vs. using the wildtype sequence. (b) AlphaFold pLDDT for each backbone template when using the designed sequence as the target sequence vs. using the wildtype sequence. (c) TM Score between the AlphaFold output structure and the target backbone when using the designed sequence as the target sequence vs. using the wildtype sequence. (d) AlphaFold composite confidence when using the designed sequence as the target sequence vs. using the wildtype sequence.

VII. MSA-FREE STRUCTURE PREDICTION

To improve AlphaFold's structure predictions without the need for MSAs, we designed a generator-discriminator pipeline that links two instances of AlphaFold. By iteratively perturbing the input sequence-matrix, the generator is used to sample decoy structures. Each generator output structure is passed to the discriminator as a template (with side chain atoms beyond Cβ masked and a sequence of gap characters). The discriminator then attempts to predict the target sequence, and its confidence is backpropagated to update the input sequence. Before backpropagation, the input sequence is initialized to the target sequence.

To make backpropagation easier, we utilized a confidence loss based on the discriminator's distogram prediction head. The entropies of AlphaFold's distance predictions convey model confidence, and using this signal as a loss does not require backpropagating through the structure module. More specifically, the loss function calculates the discriminator confidence for a given position by outputting the lowest entropy of any predicted distance distribution between that position and another residue more than 9 positions away. This entropy is calculated over the subset of bins in the distogram corresponding to distances less than 14 angstroms. Thus, the confidence loss is defined as:

```
loss = -(softmax(logits[bins<14])*
log(softmax(logits))[bins<14]).
```

This formulation assesses AlphaFold's confidence in its predictions of tertiary contacts.

We experimented with two different ways of linking the instances of AlphaFold together: 1) by passing the predicted distogram from the generator to the discriminator model as a template distogram, and 2) by passing the predicted distogram and pair representations from the generator into the discriminator via AlphaFold's recycling mechanism. Both approaches avoid backpropagating through the structure module by directly passing the predicted distogram, making optimization smoother.

For the input sequence optimization, we experimented with two approaches: 1) greedily optimizing random point mutations and 2) backpropagating the loss and updating the input sequence using stochastic gradient descent. For the first approach, at each iteration, 10 random mutations were evaluated and the mutation resulting in the minimal loss was fixed. 10 independent trajectories with 50 iterations each were performed. The discriminator output structure with the best loss across all iterations and trajectories was selected (Figure S10A, Figure S11). For the second approach, the input sequence-matrix was treated as an unconstrained set of continuous values during optimization. 50 independent trajectories were carried out for 50 steps each with a learning rate of 0.1 (Figure S10B, Figure S12). The same was performed for the recycle experiment (Figure S10C, Figure S13).

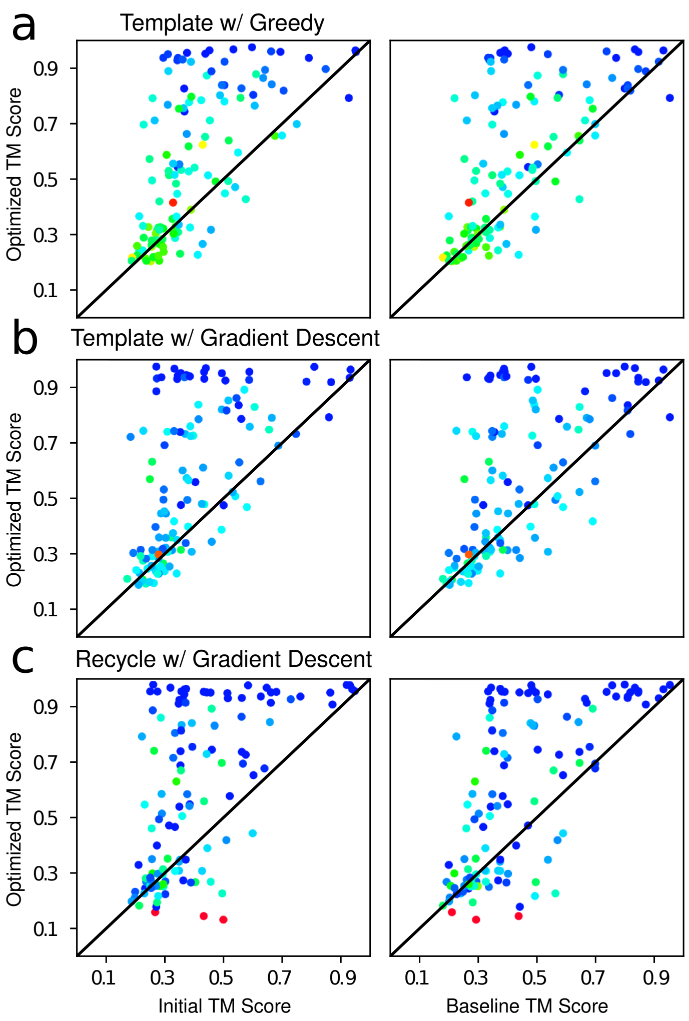

FIG. S10. Decoy generation using AlphaFold improves structure predictions from single sequences, regardless of sampling techniques. (a) Semi-greedy mutant optimization. (b) Gradient descent via the template mechanism. (c) Gradient descent via the recycling mechanism. Left column compares structure accuracy before and after optimization. Each dot is one of the 133 proteins in the Rosetta Decoy set. To control for the fact that linking two models is similar to an instance of recycling, the right column compares the predicted structure after optimization to the prediction from the baseline AlphaFold protocol with 3 recycles and a single-sequence input. The color is the discriminator pLDDT (red to blue, 40 to 90)

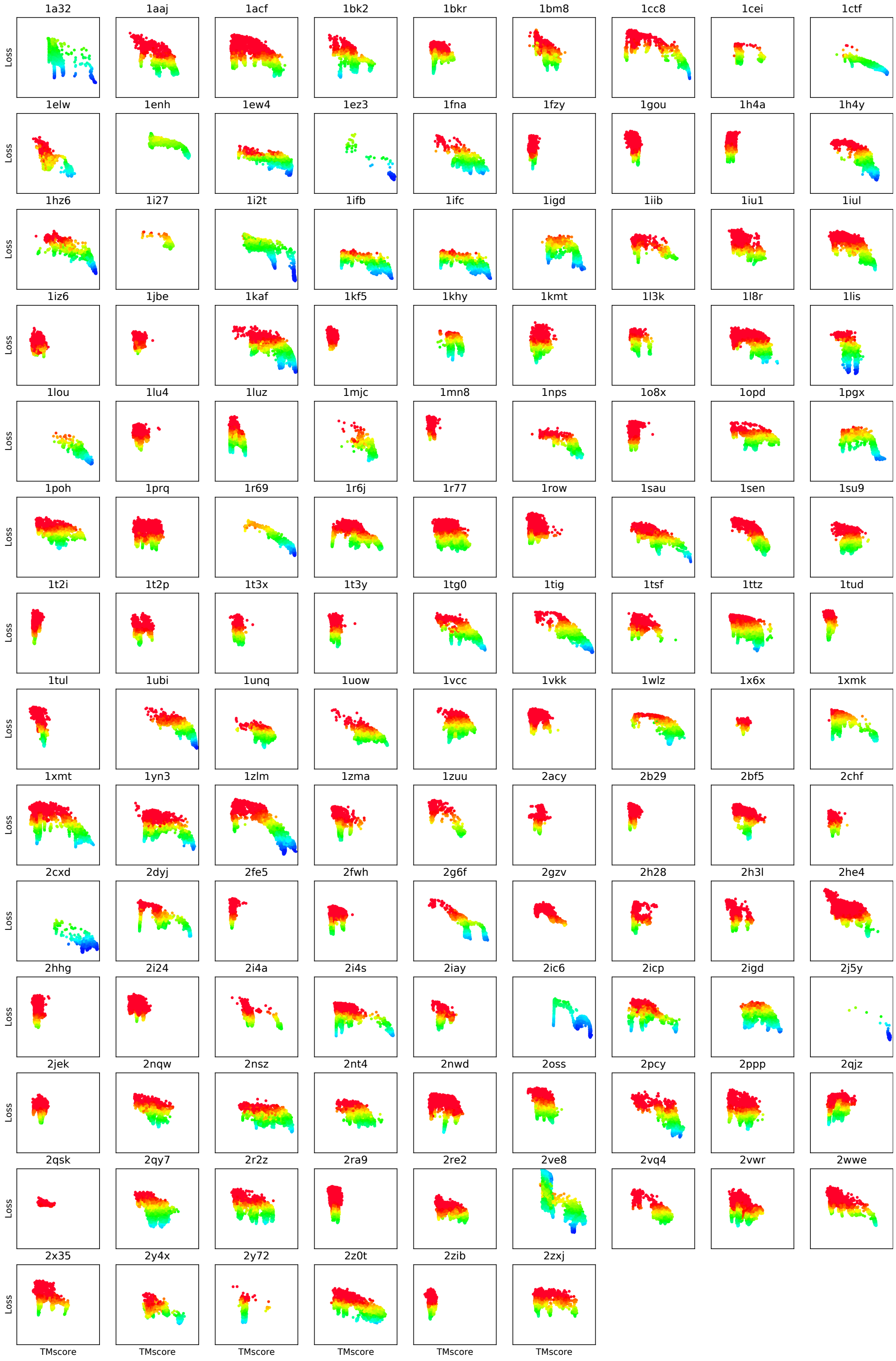

FIG. S11. Ranking AlphaFold-generated decoys with semi-greedy mutant optimization via templating mechanism. X-axis is the TMscore (range 0 to 1), y-axis is loss (range 1.4 to 4.6), color is the discriminator pLDDT (red to blue, 40 to 90)

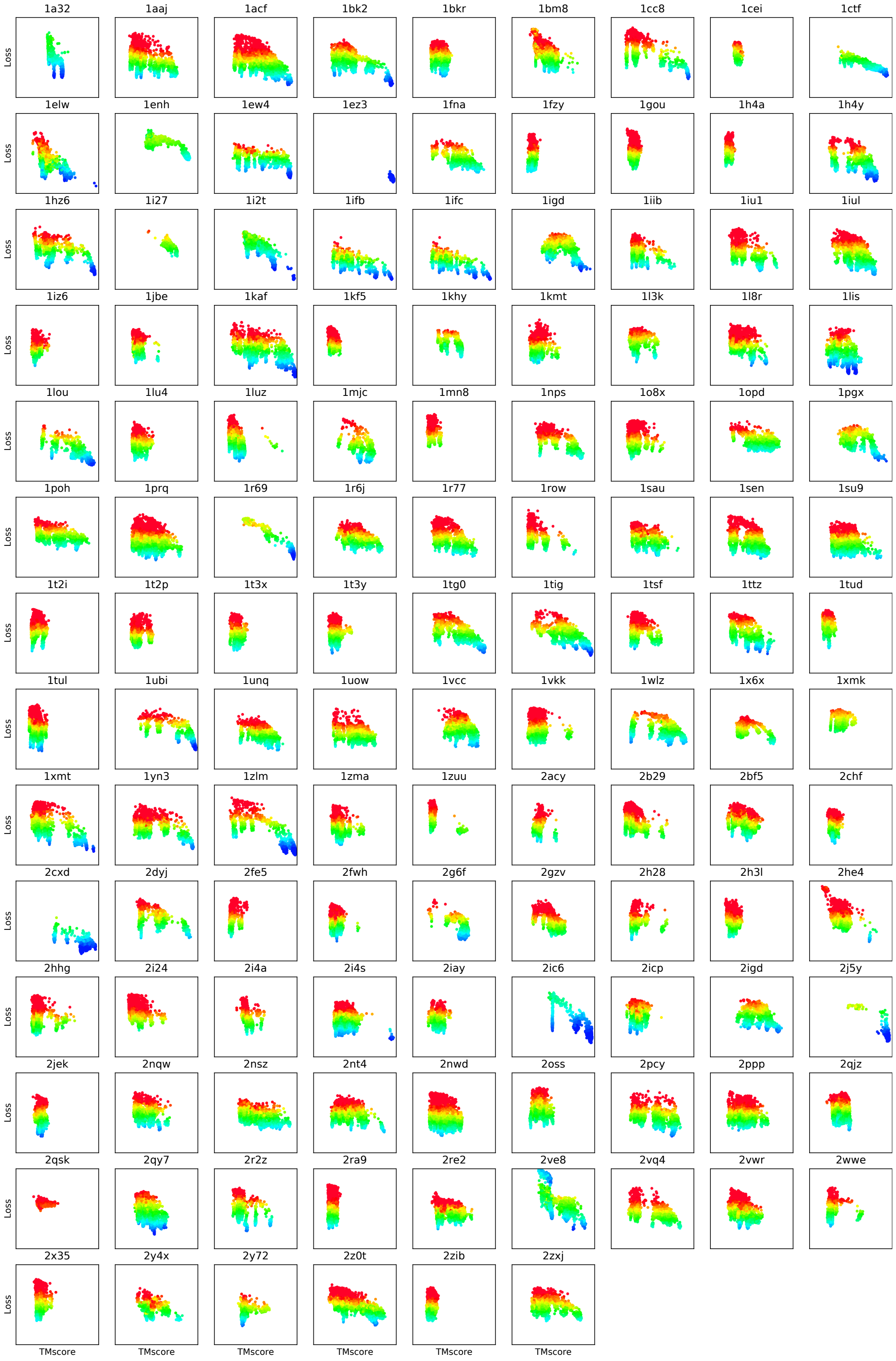

FIG. S12. Ranking AlphaFold-generated decoys with gradient descent optimization via templating mechanism. X-axis is the TMscore (range 0 to 1), y-axis is loss (range 1.4 to 4.6), color is the discriminator pLDDT (red to blue, 40 to 90)

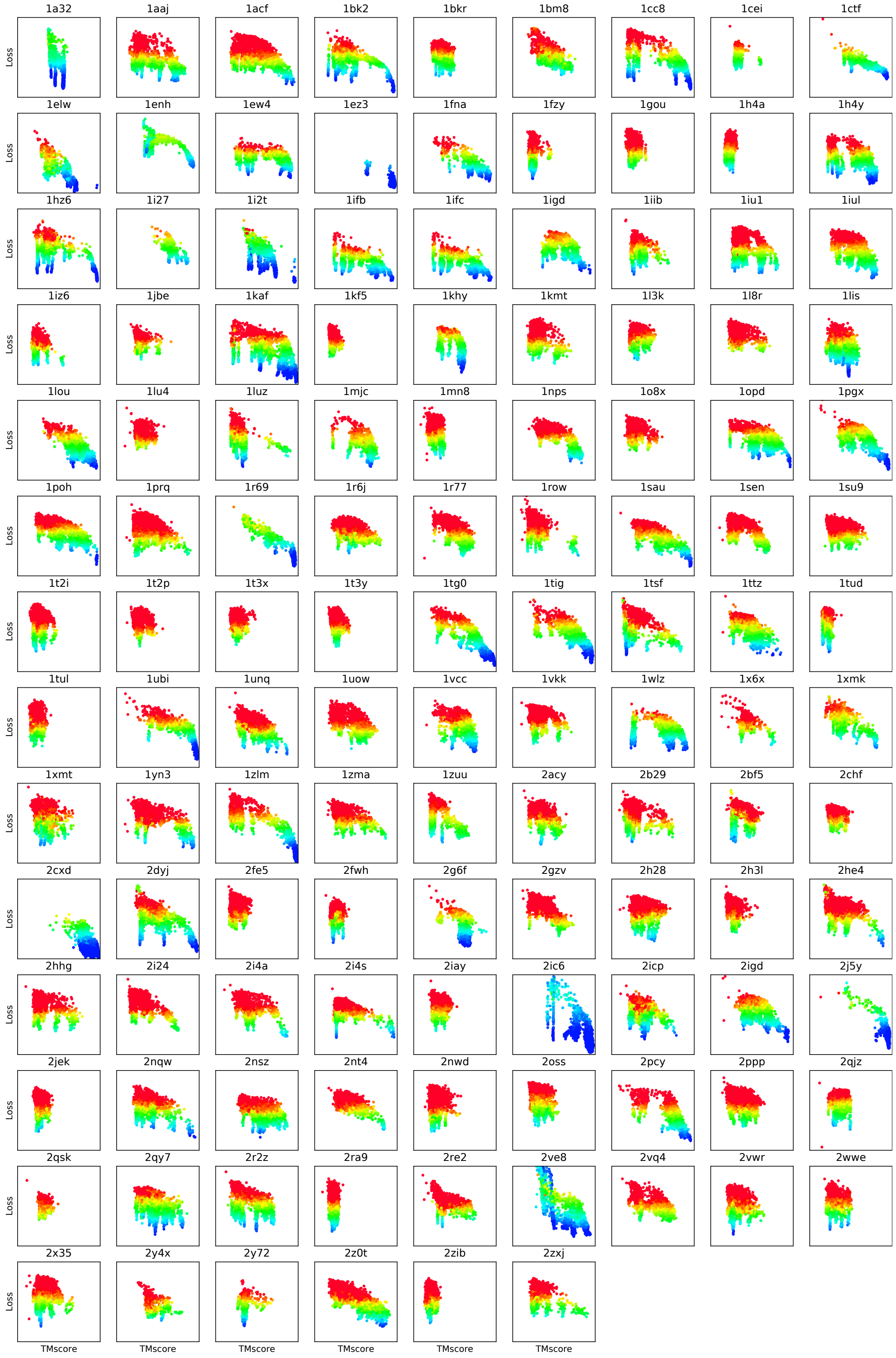

FIG. S13. Ranking AlphaFold-generated decoys with gradient descent optimization via recycling mechanism. X-axis is the TMscore (range 0 to 1), y-axis is loss (range 1.4 to 4.6), color is the discriminator pLDDT (red to blue, 40 to 90)

[23] M. A. Stiffler, D. R. Hekstra, and R. Ranganathan, Evolvability as a function of purifying selection in TEM-1 β-lactamase, Cell **160**, 882 (2015).
[24] C. D. Aakre, J. Herrou, T. N. Phung, B. S. Perchuk, S. Crosson, and M. T. Laub, Evolving new protein-protein interaction specificity through promiscuous intermediates, Cell **163**, 594 (2015).
[25] M. H. Høie, C. Matteo, A. Haagen Beck Frederiksen, and K. Stein, Amelie Lindorff-Larsen, Predicting and interpreting large-scale mutagenesis data using analyses of protein stability and conservation, Cell Reports **38** (2022).
